# Health economic evidence for adjuvant chemotherapy in stage II and III colon cancer: a systematic review

**DOI:** 10.1186/s12962-023-00422-2

**Published:** 2023-01-31

**Authors:** Yat Hang To, Peter Gibbs, Jeanne Tie, Maarten IJzerman, Koen Degeling

**Affiliations:** 1 Personalised Oncology Division, Walter and Eliza Hall Institute of Medical Research, Melbourne, Australia; 2 Department of Medical Oncology, Peter MacCallum Cancer Centre, Melbourne, Australia; 3 Department of Medical Oncology, Western Health, Melbourne, Australia; 4 Faculty of Medicine and Health Sciences, University of Melbourne, Melbourne, Australia; 5 Cancer Health Services Research, Centre for Cancer Research, Faculty of Medicine, Dentistry and Health Sciences, University of Melbourne, Melbourne, Australia; 6 Cancer Health Services Research, Centre for Health Policy, Melbourne School of Population and Global Health, Faculty of Medicine, Dentistry and Health Sciences, University of Melbourne, Melbourne, Australia; 7 Department of Cancer Research, Peter MacCallum Cancer Centre, Melbourne, Australia

**Keywords:** Colon, Cancer, Systematic, Economic, Cost-effectiveness

## Abstract

**Objective:**

The aims of this study was to appraise the health economic evidence for adjuvant chemotherapy (AC) strategies in stage II and III colon cancer (CC) and identify gaps in the available evidence that might inform further research.

**Method:**

A systematic review of published economic evaluations was undertaken. Four databases were searched and full-text publications in English were screened for inclusion. A narrative synthesis was performed to summarise the evidence.

**Results:**

Thirty-eight studies were identified and stratified by cancer stage and AC strategy. The majority (89%) were full economic evaluations considering both health outcomes, usually measured as quality-adjusted life years (QALYs), and costs. AC was found to be cost-effective compared to no AC for both stage II and III CC. Oral and oxaliplatin-based AC was cost-effective for stage III. Three months of CAPOX was cost-effective compared to 6-month in high-risk stage II and stage III CC. Preliminary evidence suggests that biomarker approaches to AC selection in stage II can reduce costs and improve health outcomes. Notably, assessment of QALYs were predominantly reliant on a small number of non-contemporary health-utility studies. Only 32% of studies considered societal costs such as travel and time off work.

**Conclusions:**

Published economic evaluations consistently supported the use of AC in stage II and III colon cancer. Biomarker-driven approaches to patient selection have great potential to be cost-effective, but more robust clinical and economic evidence is warranted. Patient surveys embedded into clinical trials may address critical knowledge gaps regarding accurate assessment of QALYs and societal costs in the modern era.

**Supplementary Information:**

The online version contains supplementary material available at 10.1186/s12962-023-00422-2.

## Background

A wide range of adjuvant (AC) strategies are currently employed in stage II and III colon cancers (CC) following surgical resection. Single-agent regimens include intravenous 5-fluorouracil (5FU) or oral equivalents such as capecitabine or tegafur-uracil (UFT). Oxaliplatin-based doublet chemotherapy, either FOLFOX (5-FU + oxaliplatin) or CAPOX (capecitabine + oxaliplatin), are standard in stage III but can also be considered in select stage II patients. For patients undergoing oxaliplatin-based chemotherapy, the results of the International Duration Evaluation of Adjuvant Chemotherapy (IDEA) collaboration suggests that the duration of oxaliplatin-based AC may be shortened from 6 to 3 months in those patients assessed to be at lower risk of recurrence based on tumour and nodal staging [1].

As CC incidence is projected to increase over time, questions surrounding the economic costs of AC strategies will become increasingly important [2, 3]. As an illustration, accounting for drug acquisition, delivery, and management of toxicities, a 6 month course of adjuvant FOLFOX chemotherapy costs in excess of 10,000 US dollars (USD) per patient [4, 5]. Strategies to reduce AC utilisation, such as shortening duration of oxaliplatin-based AC from 6 to 3 months, has been projected to result in healthcare system savings of 3.6 to 61.4 million USD over a 5 year period depending on the country [6]. Another strategy to reduce costs is the use of molecular and genomic biomarkers that allows more precise identification of patients at high risk of recurrence that would most likely benefit from AC, reducing overtreatment by avoiding AC in patients who would least benefit [7–10]. In many countries, such new technologies require reimbursement through taxpayer funds to ensure affordable access. Navigating reimbursement requires demonstration of economic value, which is usually provided by health economic evaluations. As these evaluations are usually derived from clinical trials, more efforts should be made to design trials with future reimbursement considerations in mind.

To appraise the health economic evidence of AC strategies in CC, a systematic review of published evaluations assessing AC treatment strategies in stage II and III CC was undertaken. This review also aimed to identify common assumption and limitations in published evaluations that may inform future trial designs, expediting patient access to new therapies and health technologies.

## Methods

This review was designed, performed, and reports in line with the Preferred Reporting Items of Systematic Reviews and Meta-Analyses Guidelines, and prospectively registered in the International Prospective Register of Systematic Reviews (CRD42021265063) [11, 12].

### Search strategy and study selection

The literature search was performed on 8th July 2021 using the Ovid platform to access the MEDLINE, EMBASE, Health Technology Assessment, and National Health Service Health Economic Evaluation Database platforms. The search terms utilised are presented in the Additional file 1. No restrictions were applied for the year of publication, but studies were restricted to English language only. An updated search was additionally performed on 10th December 2021.

Duplicates were removed and two reviewers independently screened titles and abstracts, followed by full text screening. Disagreement was resolved by consensus. The references of included publications were screened for further articles of interest. The full inclusion and exclusion criteria are presented in Table 1. As this analysis reviews the economic evidence for AC strategies following resection of the primary tumour, studies concerning low rectal cancers, often referred to in literature only as “rectal cancers”, have been excluded as treatment incorporates neoadjuvant radio- and chemotherapy and occasionally, avoidance of surgical resection entirely [13, 14]. In comparison, patients with colon and high rectal cancers, referred to collectively in this review as “colon cancers”, are recommended by guidelines to have upfront resection followed by consideration of AC [15, 16]. An evaluation had to meet all inclusion criteria and not fulfil any exclusion criteria to be included in the systematic review.

**Table 1:** Full inclusion and exclusion criteria used to screen prospective publications

Inclusion criteria	Exclusion criteria
1. Studies that include resected stage II and III colon cancers 2. Study intervention includes adjuvant chemotherapy or molecular/genomic biomarkers guiding adjuvant chemotherapy patient selection 3. Studies evaluating different adjuvant chemotherapy strategies such as chemotherapy vs. no chemotherapy, use of specific regimens, addition/absence of oxaliplatin and duration of chemotherapy 4. Model- or trial-based health economic evaluations such as cost–benefit and effectiveness analyses, costing studies, budget impact analyses	1. Studies that exclusively focus on rectal cancer (including neoadjuvant and/or surgical strategies) 2. Studies that includes or exclusive to other cancer types aside from colon (including anal cancer) 3. Studies pertaining to surgical strategies and/or neoadjuvant therapy 4. Studies evaluating non-consensus guideline supported adjuvant therapy or medications (including intraportal chemotherapy, adjuvant radiotherapy and chemoradiotherapy) 5. Studies pertaining to screening, prevention, surveillance and/or treatment of metastatic disease 6. Studies exclusively evaluating cost of illness 7. Studies exclusively evaluating strategies to limit or avoid toxicities 8. Studies exclusively evaluating quality of life or evaluation of health utility values without consideration of costs 9. Studies exclusively evaluating use of molecular and genomic markers without consideration of costs 10. Studies that compare hospital-based to home-based chemotherapy delivery 11. Reviews, commentaries, letters and abstracts

### Data extraction

Data including general article and clinical information, economic methods, and study outcomes was extracted from all included publications utilising a pre-defined data extraction template in Microsoft Excel. The Consolidated Health Economic Evaluation Reporting Standards (CHEERS) checklist, a set of recommendations guiding economic evaluation reporting, was adapted as a report-quality scoring tool, scoring each study according to the proportion of the applicable CHEERS items they reported [17].

## Results

**Fig. 1:**
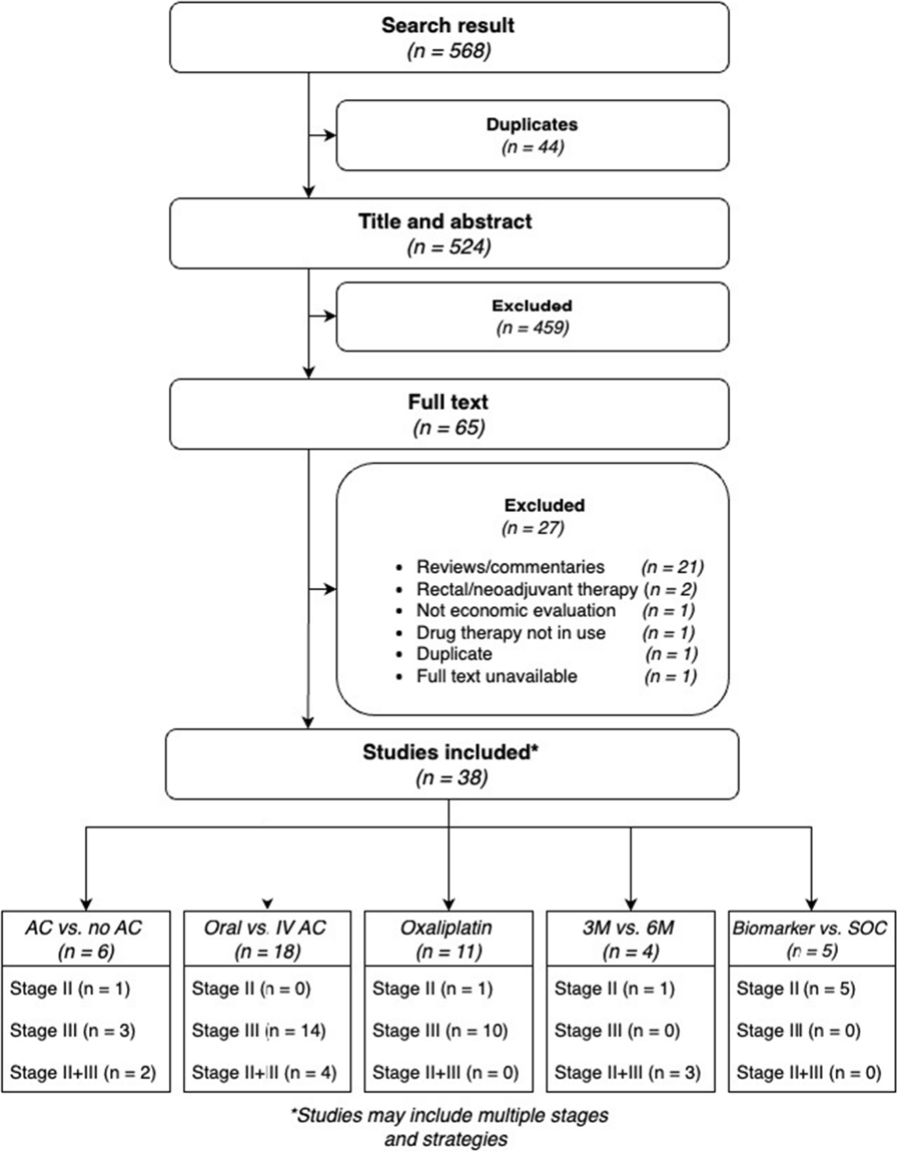
Graphical representation of the search and selection process

The initial literature search identified 568 publications, of which 44 duplicates were removed. Of the 524 unique publications, 459 were excluded following title and abstract screening (Fig. 1). The remaining 65 studies underwent full text review, with 38 meeting the inclusion criteria [4–6, 18–52]. Reasons for exclusion during the full text review included studies being reviews or commentaries, pertaining to rectal cancer or neoadjuvant therapies, not being economic evaluations or full text being unavailable. Another study was excluded as it evaluated a drug that is no longer in used in current clinical practice and therefore, the evaluation was of limited relevance. Selected studies were stratified by stage (II or III) and the AC strategies being investigated, with some studies found to examine multiple strategies or report aggregated stage II and III results.

### General characteristics

Table 2 summarises the main characteristics and key results of the identified evaluations. Where applicable, we provided the cost-effectiveness judgement reported by the author(s) and the willingness-to-pay (WTP) threshold per health outcome.

**Table 2:** Summary of the study characteristics and key findings of the final sample of economic evaluations

	Stage	Publication	Country (currency)	Economic perspective	Evaluation type	Modelling technique	Time horizon	Discount rate (%)	Treatment strategies (experimental vs. control)	Health outcomes	Impact of experimental vs. control strategy on cost	Impact of experimental vs. control strategy on health outcomes	Authors’ cost-effectiveness judgement	WTP
AC vs. no AC	II	Ayaci, 2013 [18]	USA (USD)	Healthcare payer	CUA	Markov	5 years	3	5FU vs. no AC	QALY	Increase	Increase	Cost effective	50,000
									FOLFOX vs. no AC	QALY	Increase	Increase	Not cost-effective	
	III	Smith, 1993 [19]	Australia (AUD)	Healthcare payer	CUA	Decision tree	20 years	5	5FU + LV vs. no AC	QALY	Increase	Increase	Author did not provide conclusion	Not reported
		Brown, 1994 [20]	USA (USD)	Societal	CEA	Markov	30 years	6	5FU + Leva vs. no AC	LY	Increase	Increase	Cost-effective	50,000
		Lairson, 2014 [21]	USA (USD)	Healthcare payer	CUA	Patient level data	Lifetime	3	5FU + LV vs. no AC	QALY	Increase	Increase	Cost-effective	100,000
									FOLFOX vs. no AC		Increase	Increase	Cost-effective	
	II and III	Norum, 1997 [22]	Norway (BP)	Healthcare payer	CUA	Patient level data	Lifetime	5	5FU + Leva vs. no AC	QALY	Increase	Increase	Cost effective	20,000
		Michel, 1999 [23]	France (USD)	Healthcare payer	CEA	Decision tree	5 years	No discount	AC in stage II and III vs. AC in stage III only	No. of surviving patients	Increase	Increase	Cost-effective	10,000
Oral vs. IV chemotherapy	III	Cassidy, 2006 [24]	UK (BP)	Societal	CEA, CUA	PSA	Lifetime	1.5 (cost); 6 (effect)	Capecitabine vs. 5FU	LM, QALM	Decrease	Increase	Capecitabine dominates 5FU	Not reported
		Eggington, 2006 [25]	UK (BP)	Healthcare payer	CEA, CUA	Markov	50 years	6 (cost); 1.5 (effect)	Capecitabine vs. 5FU	LY, QALY	Decrease	Increase	Capecitabine dominates 5FU	20,000
		Ho, 2006 [26]	Canada (CAD)	Societal	CMA	Decision tree	5 years	NR	XELOX vs. FOLFOX	N/A	Decrease	N/A	N/A	N/A
		Douillard, 2007 [27]	France (Euro)	Healthcare payer	CC	Decision tree	3 years	No discount	Capecitabine vs. 5FU	Relapse-free survival	Decrease	Increase	Capecitabine dominates 5FU	Not reported
		DiConstanzo, 2008 [28]	Italy (Euro)	Healthcare payer	CEA, CUA	PSA	10 years	3.5	Capecitabine vs. 5FU	LM, QALM	Decrease	Increase	Capecitabine dominates 5FU	Not reported
		Goerner, 2009 [29]	Germany (Euro)	Healthcare payer	Costing analysis	Decision tree	6 months	NR	Capecitabine vs. 5FU	N/A	Decrease	N/A	N/A	N/A
		Shiroiwa, 2009 [30]	Japan (Yen)	Healthcare payer	CUA	Markov	30 years	3	Capecitabine vs. 5FU	QALY	Decrease	Increase	Capecitabine dominates 5FU	0
		Hsu, 2011 [31]	UK (BP)	Healthcare payer	CUA	PSA	10 years	3	Capecitabine vs. 5FU	QALM	Decrease	Increase	Capecitabine dominates 5FU	Not reported
		Xie, 2013 [32]	China (USD)	Societal	Costing analysis	Patient level data	6 months	No discount	CAPOX vs. FOLFOX	N/A	Decrease	N/A	N/A	N/A
		Soni, 2014 [33]	US (USD)	Healthcare payer	CUA	Markov	5 years	3	Capecitabine vs. 5FU	QALY	Increase	Decrease	5FU dominates Capecitabine	100,000
		Chen, 2015 [34]	Taiwan (NT)	Societal	CUA	Patient level data	28 weeks	No discount	Capecitabine ± oxaliplatin vs. 5FU ± oxaliplatin	Health-related QOL scores	Decrease	No difference	Cost-effective	Not reported
		Lerdkiattikorn, 2015 [35]	Thailand (Baht)	Societal	CUA	Markov	99 years	3	Capecitabine vs. 5FU	QALY	Increase	Increase	Not cost effective	300,000
		Lin, 2015 [36]	Taiwan (NT)	Societal	Costing analysis	Patient level data	25 months	No discount	Capecitabine vs. 5FU	Health-related QOL scores	Decrease	No difference	Cost saving	N/A
		vanGils, 2015 [37]	Netherlands (Euro)	Healthcare sector	Costing analysis	Patient level data	6 months	No discount	Capecitabine vs. 5FU	N/A	Decrease	N/A	N/A	N/A
	II and III	Murad, 1997 [38]	Brazil & Argentina (Real)	Healthcare payer	CMA	Decision Tree	18 months	NR	UFT + LV vs. 5FU + LV	N/A	Decrease	N/A	N/A	N/A
		Manidakis, 2009 [39]	Greece (Euro)	Societal	CMA	Patient level data	12 months	NR	CAPOX vs. FOLFOX	N/A	Decrease	N/A	N/A	N/A
		Wen, 2014 [40]	China (USD)	Societal	CUA	Markov	6 months	NR	CAPOX vs. FOLFOX	QALY	Decrease	Decrease	Cost-effective	17,815 (3 × GDP)
		Hsu, 2019 [41]	Taiwan (USD)	Healthcare payer	CMA	Decision Tree	6 months	NR	UFT + LV vs. 5FU + LV	N/A	Decrease	Increase	N/A	N/A
Oxaliplatin vs. no oxaliplatin	II	Ayaci,, 2013 [18]	USA (USD)	Healthcare payer	CUA	Markov	5 years	3	FOLFOX vs. 5FU + LV	QALY	Increase	Increase	Not cost effective	50,000
	III	Pandor, 2006 [42]	UK (BP)	Healthcare payer	CEA, CUA	Markov	50 years	6 (cost); 1.5 (effect)	FOLFOX vs. 5FU + LV	QALY	Increase	Increase	Cost-effective	20,000
									FOLFOX vs. 5FU + LV		Increase	Increase	Cost-effective	
									FOLFOX vs. Capecitabine		Increase	Increase	Cost-effective	
		Eggington, 2006 [25]	UK (BP)	Healthcare payer	CEA, CUA	Markov	50 years	6 (cost); 1.5 (effect)	FOLFOX vs. 5FU + LV	LY, QALY	Increase	Increase	Cost-effective	20,000
		Aballea, 2007 [43]	UK (BP)	Healthcare payer	CUA	PSA	50 years	3.5	FOLFOX vs. 5FU + LV	QALY	Increase	Increase	Cost effective	30,000
		Aballea, 2007 [44]	USA (USD)	Healthcare payer	CUA	PSA	50 years	3	FOLFOX vs. 5FU + LV	QALY	Increase	Increase	Cost effective	50 – 100,000
		Goerner, 2009 [29]	Germany (Euro)	Healthcare payer	Costing analysis	Decision tree	6 months	No discount	FOLFOX vs. 5FU + LV	N/A	Increase	N/A	N/A	N/A
									CAPOX vs. 5FU + LV		Increase	N/A	N/A	
		Attard, 2010 [45]	Canada (CAD)	Healthcare payer	CUA	PSA	50 years	5	FOLFOX vs. 5FU + LV	QALY	Increase	Increase	Cost-effective	Not reported
		Shiroiwa, 2012 [46]	Japan (Yen)	Healthcare payer	CUA	PSA	30 years	3	FOLFOX vs. 5FU + LV	QALY	Increase	Increase	Cost-effective	5 million
		Soni, 2014 [33]	USA (USD)	Healthcare payer	CUA	Markov	5 years	3	FOLFOX vs. 5FU + LV	QALY	Increase	Increase	Cost-effective	100,000
									CAPOX vs. 5FU + LV	QALY	Increase	Decrease	5FU dominates CAPOX	100,000
		Lerdkiattikorn, 2015 [35]	Thailand (Baht)	Societal	CUA	Markov	99 years	3	FOLFOX vs. 5FU + LV	QALY	Increase	Increase	Not cost effective	300,000
		vanGils, 2015 [37]	Netherlands (Euro)	Healthcare sector	Costing analysis	Patient level data	6 months	NR	FOLFOX vs. 5FU + LV	N/A	Increase	N/A	N/A	N/A
									FOLFOX vx. Capecitabine	N/A	Increase	N/A	N/A	
									CAPOX vs. 5FU + LV	N/A	Increase	N/A	N/A	
									CAPOX vs. Capecitabine	N/A	Increase	N/A	N/A	
3 M vs. 6 M	II	Jongeneel, 2020 [4]	Netherlands (Euro)	Societal	CUA	Markov	Lifetime	4 (cost); 1.5 (effect)	3 M vs. 6 M FOLFOX	QALY	Decrease	Decrease	Not cost-effective; negative NMB	50,000
									3 M vs. 6 M CAPOX		Decrease	Increase	3 M CAPOX dominates 6 M	
	II and III	Robles-Zurita, 2018 [47]	UK (BP)	Healthcare sector	CUA	PSA	8 years	3.5	3 M vs. 6 M CAPOX	QALY	Decrease	Increase	3 M dominates 6 M	30,000
		Iveson, 2019 [48]	UK (BP)	Healthcare sector	CUA	PSA	8 years	3.5	3 M vs. 6 M AC	QALY	Decrease	Increase	3 M dominates 6 M	30,000
		Hanna, 2021 [6]	Multi-country (USD)	Healthcare sector	CUA, BIA	Patient level data	10 year	3.5	3 M vs. 6 M AC	QALY	Decrease	Increase	Cost effective	42,000
Biomarker	II	Hornberger, 2012 [49]	USA (USD)	Societal	CUA	Markov	Lifetime	3	Oncotype Dx vs. SOC	QALY	Decrease	Increase	Genomic assay dominates SOC	50,000
		Alberts, 2014 [50]	USA (USD)	Healthcare payer	CUA	Markov	Lifetime	3	OncotypeDx vs.SOC	QALY	Decrease	Increase	Genomic assay dominates SOC	50,000
		Jongeneel, 2021 [51]	Netherlands (Euros)	Societal	CUA	Markov	Lifetime	4 (cost); 1.5 (effect)	Biomarker (MSS + BRAF/KRAS) vs. SOC	QALY	Increase	Increase	Cost-effective	50,000
		To, 2021 [5]	Australia (AUD)	Healthcare payer	CUA	Markov	Lifetime	5	ctDNA vs. SOC	QALY	Decrease	Increase	ctDNA dominate SOC	20,000
		Alarid-Escuder, 2021 [52]	USA (USD)	Healthcare payer	CUA	Markov	Lifetime	3	Biomarker (CDX2) vs. no AC	QALY	Increase	Increase	Cost-effective	100,000

*3 M* 3 month duration of chemotherapy, *6 M* 6 month duration of chemotherapy, *5FU* 5-fluorouracil, *AC* adjuvant chemotherapy, *AUD* Australian Dollars, *BIA* Budget impact analysis, *BP* British Pound, *CEA* cost-effectiveness analysis, *CMA* cost-minimisation analysis, *ctDNA* circulating tumour DNA, *CUA* cost-utility analysis, *IV* intravenous, *LV* leucovorin, *LY* life-years, *PSA* partitioned survival analysis, *NT* Taiwan Dollar, *NR* not reported, *QALY* Quality-adjusted life years, *SOC* standard of care, *USD* United State Dollars

Of the 38 studies, 34 (89%) were full economic evaluations with 22 cost-utility analysis (CUA), 4 cost-minimisation analysis (CMA), 2 cost-effectiveness analysis (CEA), 4 combined CUA and CEA, 1 combined CUA and budget impact analysis, and 1 cost-consequence (CC) study. There were 4 partial evaluations that only considered costs.

### Perspective and costs

Regarding the perspective on costs, 21 studies (55%) adopted a healthcare payer perspective, 12 (32%) a societal perspective, which considers additional patient-related costs, and 5 (13%) a healthcare sector perspective.

All studies considered the AC drugs and administration costs, 29 (76%) considered costs from adverse events (AEs) that did not result in hospitalisation, 33 (87%) hospitalisations from AEs, 23 (61%) cancer surveillance, 21 (55%) treatment of recurrence, 12 (32%) patient travel costs, and 13 (34%) costs due to loss of productivity.

Of the studies that costed adverse events (n = 33), 21 (64%) specifically costed the management of treatment-related febrile neutropenia, 21 (64%) diarrhoea, and 19 (58%) nausea and vomiting. Twelve studies (36%) considered the cost of additional AEs with the most common being oxaliplatin-related neurotoxicity (8 of 24 studies that included oxaliplatin), stomatitis/mucositis (n = 8) and palmar-plantar erythrodysesthesia (PPE; n = 6).

### Health utility values

The most common measure of health outcomes were quality-adjusted life years (QALYs), reported by 27 economic evaluations. In measuring QALYs, 21 (78%) of these studies utilised health utility values (HUVs) from literature with the remaining measuring HUVs directly from recruited patients. Nineteen of the evaluations relied on published HUV utilised values measured in the 1990s [53, 54]. Notably, all studies comparing AC duration adopted utilities derived from patient surveys collected alongside the SCOT study, a phase III clinical trial randomising patients to 3 or 6 months AC [55].

### Quality of reporting

On average, the evaluations reported 89% of applicable items on the CHEERS checklist (range: 74% to 100%). The mostly poorly reported CHEERS items were the abstract (58%) due to incomplete reporting of uncertainty analyses, model parameters (63%), and conflict of interest (63%). The detailed scoring is available in the Additional file 1.

### AC vs. no AC

All studies reported that AC was cost-effective compared to no AC in stage II (n = 3) and stage III (n = 5) [18–23]. All studies only considered single agent AC aside from one study that concluded doublet AC was cost-effective in stage III patients [21].

### Oral vs. intravenous AC

Of the 9 full economic evaluations comparing capecitabine to 5FU in stage III, 6 (67%) concluded capecitabine dominates 5-FU (being less costly and more effective) and 1 (11%) reported cost-effectiveness (more costly but more effective) [24–32, 34, 36–41]. Of the two remaining studies, Soni et al. utilised data from a retrospective cohort study, noting patients receiving capecitabine were older (mean age: 73 vs. 67 years) and less fit (ECOG 2–4: 14.6% vs. 6.3%) [33]. Accordingly, these patients were less likely to receive full intensity of treatment with the authors concluding that capecitabine would be cost-effective if treatment intensity approached 100%. In the remaining study, the cost of capecitabine acquisition was reported as almost 10 times the cost of 5-FU, which is substantially higher than reported in other studies [35]. Notably, there was no full evaluation comparing capecitabine to 5FU in stage II CC.

Further analyses demonstrated that CAPOX reduced costs compared to FOLFOX with one CUA reported cost-effectiveness in a combined cohort of high-risk stage II and stage III patients [26, 34, 39, 40].

### Oxaliplatin-based AC

There was only one evaluation of oxaliplatin-based therapy in stage II patients, concluding that FOLFOX was not cost-effective compared to 5-FU [18]. Amongst stage III studies, 7 of 8 (88%) full economic evaluations determined that FOLFOX was cost-effective compared to 5-FU alone [25, 33, 42–46]. The remaining study modelled strategies that included different treatments for metastatic recurrence, limiting the assessment of AC alone [35].

Additionally, Pandor et al. concluded that FOLFOX dominates capecitabine in stage III [42]. Conversely, Soni et al. concluded that 5-FU dominates CAPOX but as previously noted, capecitabine-treated patients in this study were less likely to receive the full intensity of treatment, limiting efficacy [33].

### Three vs. six month duration

Three studies were modelled on data from the SCOT trial, a randomised controlled trial of 3 versus 6 months of oxaliplatin-based doublet AC in a cohort of high-risk stage II and stage III patients [4, 6, 47, 48]. Two of these studies concluded that 3 months of AC dominates 6 months. The remaining study assumed partial prescription of shortened AC duration based on a survey of physicians (stage II: 18%; stage III: 50%) but despite this limited uptake, a 3-month treatment duration was still cost-effective [6].

Jongeneel et al. analysed AC in high-risk stage II patients by specific regimen, concluding that 3 months of CAPOX dominates 6 months but that 3 months of FOLFOX was not cost-effective [4]. Importantly, they considered T4 staging and microsatellite stability (MSS) as high-risk histological features compared to the more expansive definition of high risk disease utilised in the SCOT trial [55].

### Biomarker vs. standard of care

Two studies evaluated the use of OncotypeDx, a clinically validated and commercially available tumour-based genomic assay, in stage II patients with T3 and pMMR tumours. Compared to SOC patient selection, both studies reported an absolute decrease in AC prescription based on assay use (17–22%), concluding that Oncotype Dx reduces cost whilst improving health outcomes [49, 50].

To et al. modelled the use of post-operative circulating tumour DNA (ctDNA) in unselected stage II CC patients. Assigning AC to patients with detectable ctDNA alone resulted in a 13% absolute reduction in AC prescription compared to SOC [5]. Furthermore, they considered a scenario in which some ctDNA negative patients would also receive AC. Both complete and incomplete adherence to ctDNA testing resulted in ctDNA dominating SOC [5].

Jongeneel et al. compared several AC selection strategies in stage II cancers that were T4, and MSS tumours utilising a biomarker approach based on the presence of BRAF and KRAS mutations [51]. The study concluded that AC prescription based on molecular biomarkers (4.8%) dominates no patients receiving AC. Patients in this model could have received either 3-months of CAPOX or 6-months of 5FU or FOLFOX, with the biomarker strategy retaining cost-effectiveness in scenarios in which only capecitabine-based AC was prescribed.

Alarid-Escudero et al. modelled a biomarker approach in stage II patients with T3 tumours, assigning FOLFOX to CDX2 negative patients only (7.2% of tested patients) [52]. Compared to no patients receiving AC, this biomarker approach was cost-effective in the base-case scenario and in > 88% of scenarios in which the effectiveness of AC was varied.

## Discussion

This systematic review included 38 health economic evaluations that compared a number of AC strategies currently employed in stage II and III CC, aiming to report the cost-effectiveness of these strategies and identify areas of potential improvements to inform further trial design and economic evaluations.

Firstly, the evaluations consistently implied that single agent AC is cost-effective compared to no AC. Secondly, most studies reported that oral capecitabine was cost-effective to or dominates (i.e., improved health outcomes at lower cost) 5-FU. In contrast, Soni et al. reported that 5FU dominates both capecitabine and CAPOX. However, this study was modelled on a real-world patient cohort, observing that as older and less fit patients were more likely to receive oral AC, capecitabine-based therapy was also associated with reduced dose intensity (RDI) and a lower probability of 5-year OS [56]. As comparison, an age based analysis of the phase III X-ACT trial reported similar efficacy of capecitabine in all age groups (including ≥ 70 years) despite higher rates of toxicity and dose reduction observed amongst older patients, contradicting the findings noted by Soni et al. [57] Economic evaluations are often based on clinical trials which recruits participants that tend to be younger and fitter than real-world counterparts, resulting in an observable difference between the efficacy observed in-trial and in routine clinical-practice [58, 59]. Modelling purely based on trial data may over-estimate efficacy but also underestimate toxicities, leading to favourable incremental cost-effective ratios (ICERs). One possible solution is the utilisation of real-world data (RWD) to inform model parameters in economic evaluations. RWD could be used to more accurately model control or standard strategies, with relative outcomes from clinical trials being applied. In the case of targeted therapies, RWD could also provide more accurate estimates of uptake based on prevalence of targeted mutations if routinely tested. Well maintained cancer registries could provide robust RWD and improve the generalisability of economic evaluation results. Notably, three of the included studies utilised such registries in modelling transitions between health states such as disease free to recurrence or death. Two studies utilised the Netherlands Cancer Registry (NCR) to develop a health model that simulates patients with stage II colon cancer from diagnosis to death [4, 51]. The third study utilised an Australian-based multi-site colorectal cancer registry (ACCORD) to model the progression of patients following recurrence to death [5]. These studies illustrates the feasibility of using RWD to inform model parameters, perhaps better reflecting real-world outcomes.

The majority of economic evaluations also reported 6-months of FOLFOX as being cost-effective compared to 5-FU in stage III CC. However, following publication of the IDEA collaboration, consensus guidelines now recommend for patients undergoing oxaliplatin-based AC, 3 months duration can also be considered based on further risk stratification [15, 16]. Given all identified economic evaluations demonstrated that 3 months of oxaliplatin-based AC dominates 6 months in stage II and III, shortened AC should be incorporated as a comparator in all economic evaluation as modelling 6 months alone may overestimate costs. Of the 2 biomarker studies published following the IDEA collaboration, only one modelled shortened CAPOX chemotherapy. Additionally, only a small number of oxaliplatin-related evaluations costed or considered long-term disutility from neurotoxicity. Given that the key rationale for shortening oxaliplatin exposure is to reduce neurotoxicity, more consistent consideration of this AE is required [1]. Economic evaluations must be careful in utilising relevant comparators in their assessments as regulators may reject reimbursement submissions that do not provide evidence for cost-effectiveness compared to current standard of care practices. Furthermore, detailed consideration of relevant toxicities and associated costs may more accurately reflect economic benefits.

Current consensus guidelines recommend the use of clinicopathological parameters to guide selection of stage II CC patients for AC. Such an approach results in 20–30% of patients receiving AC but meta-analyses reports only modest reductions in absolute recurrence risk suggesting that a significant proportion of patients are exposed to unnecessary treatment [60–63]. This has led to the significant interest in risk-stratifying biomarkers that could better define patients that would benefit from AC. We identified 5 studies that investigated a molecular or genomic biomarker approach to patient selection for stage II CC. It is noted that these approaches and their associated costs vary significantly from study to study. Additionally, the modelled efficacy of these biomarkers are based on either retrospective or early phase studies meaning the economic results are to be considered only as early indications of potential cost-effectiveness. Certainly, economic evaluations based on results from larger prospective studies such as the recently published DYNAMIC study, which randomised stage II CC patients to either a ctDNA guided approach to AC patient selection or SOC, is required to confirm cost-effectiveness [64]. Acknowledging these limitations, three of the studies demonstrated that a reduction in AC prescription (range: 14% to 22%) resulted in biomarker informed care dominating SOC, demonstrating the positive economic impact of reducing AC overtreatment [5, 49, 50]. The two remaining studies reported that compared to no AC, biomarker patient selection was cost-effectiveness but notably in these analyses, the uptake of AC in the biomarker strategy was limited (range: 5.8–7.4%). However, as a biomarker’s impact depends on whether it actually can change treatment decisions, economic evaluations should model compliance to biomarker results but only two studies did so. Alberts et al. utilised a survey of clinicians presented with OncotypeDx results and To et al. assumed a low non-compliance rate based on experiences in breast cancer patients [5, 50]. In assessing the economic value of 3 months AC, Hanna et al. utilised a clinician survey to model the clinical uptake of shortened AC, recognising significant variation in clinical practice [6]. Whilst clinician surveys have limitations, they can offer timely insight into the real-world uptake of new biomarkers or treatment recommendations, allowing more robust economic evaluations.

Most studies utilised HUVs from literature, however two of the sources were based on surveys of patients in the 1990s, which may be lower than contemporary values given improvements in the recognition and management of toxicities. Given that cost-utility analysis requires accurate estimation of QALYs, HUVs that are temporally and geographically relevant to the patient cohort under investigation are important. The SCOT study, a randomised phase III trial comparing 3–6 months AC, routinely collected quality-of-life (QoL) questionnaires from recruited patients. The QoL data formed the basis of the HUVs utilised by all economic evaluations assessing shortened duration AC, allowing for more accurate modelling of QALYs, reflecting the outcomes of patients exposed to the treatment under evaluation in a contemporary time period. This demonstrates the importance of collecting patient reported outcomes and QoL data from ongoing clinical trials with the SCOT study serving as a case study. This will allow economic evaluations to provide stronger cost-effectiveness evidence and hopefully expedite reimbursement processes. Additionally, patient surveys can also more accurately capture additional information such as travel costs and patient and carer time-off work, allowing for greater insight into societal costs.

## Conclusion

The available health economic evidence suggests that single-agent AC is cost-effective compared to no AC for both stage II and stage III CC, that oral AC is cost-effective compared to intravenous AC for stage III CC, that oxaliplatin-based AC is cost-effective for stage III CC, and that 3-month CAPOX is cost-effective compared to 6-months for high-risk stage II and low-risk stage III CC. The early evidence also suggests that biomarker-driven approaches to refine patient treatment selection for AC for stage II CC may be cost-effective compared to current standard of care, though more robust randomised clinical and economic evidence is warranted. Finally, to further increase the value of future economic evaluations and expedite access to new therapies, clinician and patient surveys should be incorporated into trials to address critical knowledge gaps and improve the robustness of health economic models.

## Supplementary Information

**Additional file 1.** Supplementary Material 1.

## Data Availability

The datasets used and/or analysed during the current study are available from the corresponding author on reasonable request.

## References

[1] Grothey A, Sobrero AF, Shields AF, Yoshino T, Paul J, Taieb J (2018). Duration of adjuvant chemotherapy for stage III colon cancer. N Engl J Med.

[2] Araghi M, Soerjomataram I, Jenkins M, Brierley J, Morris E, Bray F (2019). Global trends in colorectal cancer mortality: projections to the year 2035. Int J Cancer.

[3] Arnold M, Sierra MS, Laversanne M, Soerjomataram I, Jemal A, Bray F (2017). Global patterns and trends in colorectal cancer incidence and mortality. Gut.

[4] Jongeneel G, Greuter MJE, van Erning FN, Koopman M, Vink GR, Punt CJA (2020). Model-based evaluation of the cost effectiveness of 3 versus 6 months' adjuvant chemotherapy in high-risk stage II colon cancer patients. Therap Adv Gastroenterol.

[5] To YH, Degeling K, Kosmider S, Wong R, Lee M, Dunn C (2021). Circulating tumour DNA as a potential cost-effective biomarker to reduce adjuvant chemotherapy overtreatment in stage II colorectal cancer. Pharmacoeconomics.

[6] Hanna CR, Robles-Zurita JA, Briggs A, Harkin A, Kelly C, McQueen J (2021). Three versus six months of adjuvant doublet chemotherapy for patients with colorectal cancer: a multi-country cost-effectiveness and budget impact analysis. Clin Colorectal Cancer.

[7] Tie J, Wang Y, Tomasetti C, Li L, Springer S, Kinde I (2016). Circulating tumor DNA analysis detects minimal residual disease and predicts recurrence in patients with stage II colon cancer. Sci Transl Med..

[8] Tie J, Cohen JD, Wang Y, Christie M, Simons K, Lee M (2019). Circulating tumor DNA analyses as markers of recurrence risk and benefit of adjuvant therapy for stage III colon cancer. JAMA Oncol.

[9] Clark-Langone KM, Wu JY, Sangli C, Chen A, Snable JL, Nguyen A (2007). Biomarker discovery for colon cancer using a 761 gene RT-PCR assay. BMC Genomics.

[10] Gray RG, Quirke P, Handley K, Lopatin M, Magill L, Baehner FL (2011). Validation study of a quantitative multigene reverse transcriptase-polymerase chain reaction assay for assessment of recurrence risk in patients with stage II colon cancer. J Clin Oncol.

[11] Page MJ, McKenzie JE, Bossuyt PM, Boutron I, Hoffmann TC, Mulrow CD (2021). The PRISMA 2020 statement: an updated guideline for reporting systematic reviews. BMJ.

[12] Prospero. A systematic review of health economic evidence for adjuvant chemotherapy in stage II and III colorectal cancer https://www.crd.york.ac.uk/prospero/display_record.php?RecordID=265063: National Institute for Health Research; 2021

[13] Glynne-Jones R, Wyrwicz L, Tiret E, Brown G, Rödel C, Cervantes A (2017). Rectal cancer: ESMO Clinical Practice Guidelines for diagnosis, treatment and follow-up. Ann Oncol.

[14] Benson AB, Venook AP, Al-Hawary MM, Arain MA, Chen YJ, Ciombor KK (2020). NCCN Guidelines insights: rectal cancer, Version 6. 2020. J Natl Compr Canc Netw..

[15] Argilés G, Tabernero J, Labianca R, Hochhauser D, Salazar R, Iveson T (2020). Localised colon cancer: ESMO Clinical Practice Guidelines for diagnosis, treatment and follow-up 2020. Ann Oncol.

[16] Benson AB, Venook AP, Al-Hawary MM, Arain MA, Chen Y-J, Ciombor KK (2021). Colon Cancer, Version 2.2021, NCCN Clinical Practice Guidelines in Oncology. J Nat Comprehen Cancer Netw..

[17] Husereau D, Drummond M, Petrou S, Carswell C, Moher D, Greenberg D (2013). Consolidated health economic evaluation reporting standards (CHEERS) statement. BMJ Br Med J.

[18] Ayvaci MU, Shi J, Alagoz O, Lubner SJ (2013). Cost-effectiveness of adjuvant FOLFOX and 5FU/LV chemotherapy for patients with stage II colon cancer. Med Decis Making.

[19] Smith RD, Hall J, Gurney H, Harnett PR (1993). A cost-utility approach to the use of 5-fluorouracil and levamisole as adjuvant chemotherapy for Dukes' C colonic carcinoma. Med J Aust.

[20] Brown ML, Nayfield SG, Shibley LM (1994). Adjuvant therapy for stage III colon cancer: economics returns to research and cost-effectiveness of treatment. JNCI J Nat Cancer Inst..

[21] Lairson DR, Parikh RC, Cormier JN, Chan W, Du XL (2014). Cost-utility analysis of chemotherapy regimens in elderly patients with stage III colon cancer. Pharmacoeconomics.

[22] Norum J, Vonen B, Olsen JA, Revhaug A (1997). Adjuvant chemotherapy (5-fluorouracil and levamisole) in Dukes' B and C colorectal carcinoma. A cost-effectiveness analysis. Ann Oncol.

[23] Michel P, Merle V, Chiron A, Ducrotte P, Paillot B, Hecketsweiler P (1999). Postoperative management of stage II/III colon cancer: a decision analysis. Gastroenterology.

[24] Cassidy J, Douillard JY, Twelves C, McKendrick JJ, Scheithauer W, Bustová I (2006). Pharmacoeconomic analysis of adjuvant oral capecitabine vs intravenous 5-FU/LV in Dukes' C colon cancer: the X-ACT trial. Br J Cancer.

[25] Eggington S, Tappenden P, Pandor A, Paisley S, Saunders M, Seymour M (2006). Cost-effectiveness of oxaliplatin and capecitabine in the adjuvant treatment of stage III colon cancer. Br J Cancer.

[26] Ho MY, Chang AY, Ruan JY, Cheung WY (2016). Population-based cost-minimization analysis of CAPOX versus modified FOLFOX6 in the adjuvant treatment of stage III colon cancer. Clin Colorectal Cancer.

[27] Douillard JY, Tilleul P, Ychou M, Dufour P, Perrocheau G, Seitz JF (2007). Cost consequences of adjuvant capecitabine, Mayo Clinic and de Gramont regimens for stage III colon cancer in the French setting. Oncology.

[28] Di Costanzo F, Ravasio R, Sobrero A, Bertetto O, Vinante O, Luppi G (2008). Capecitabine versus bolus fluorouracil plus leucovorin (folinic acid) as adjuvant chemotherapy for patients with Dukes' C colon cancer: economic evaluation in an Italian NHS setting. Clin Drug Investig.

[29] Goerner M, Riemer-Hommel P (2009). Economic impact of alternative adjuvant chemotherapy regimens for stage III colon cancer. Onkologie.

[30] Shiroiwa T, Fukuda T, Shimozuma K, Ohashi Y, Tsutani K (2009). Cost-effectiveness analysis of capecitabine compared with bolus 5-fluorouracil/l-leucovorin for the adjuvant treatment of colon cancer in Japan. Pharmacoeconomics.

[31] Hsu TC, Chen HH, Yang MC, Wang HM, Chuang JH, Jao SW (2011). Pharmacoeconomic analysis of capecitabine versus 5-fluorouracil/leucovorin as adjuvant therapy for stage III colon cancer in Taiwan. Value Health.

[32] Xie Q, Wen F, Wei YQ, Deng HX, Li Q (2013). Cost analysis of adjuvant therapy with XELOX or FOLFOX4 for colon cancer. Colorectal Dis.

[33] Soni A, Aspinall SL, Zhao X, Good CB, Cunningham FE, Chatta G (2014). Cost-effectiveness analysis of adjuvant stage III colon cancer treatment at veterans affairs medical centers. Oncol Res.

[34] Chen HH, Chen WT, Lee HC, Lin JK, Fang CY, Chou YH (2015). Health-related quality of life and cost comparison of adjuvant capecitabine versus 5-fluorouracil/leucovorin in stage III colorectal cancer patients. Qual Life Res.

[35] Lerdkiattikorn P, Chaikledkaew U, Lausoontornsiri W, Chindavijak S, Khuhaprema T, Tantai N (2015). Cost-utility analysis of adjuvant chemotherapy in patients with stage III colon cancer in Thailand. Expert Rev Pharmacoecon Outcomes Res.

[36] Lin JK, Tan EC, Yang MC (2015). Comparing the effectiveness of capecitabine versus 5-fluorouracil/leucovorin therapy for elderly Taiwanese stage III colorectal cancer patients based on quality-of-life measures (QLQ-C30 and QLQ-CR38) and a new cost assessment tool. Health Qual Life Outcomes.

[37] van Gils CW, de Groot S, Tan SS, Redekop WK, Koopman M, Punt CJ (2015). Real-world resource use and costs of adjuvant treatment for stage III colon cancer. Eur J Cancer Care (Engl).

[38] Murad A, de Andrade CA, Delfino C, Arikian S, Doyle J, Sinha N (1997). A pharmacoeconomic comparison of UFT and 5-FU chemotherapy for colorectal cancer in South America. Oncology (Williston Park).

[39] Maniadakis N, Fragoulakis V, Pectasides D, Fountzilas G (2009). XELOX versus FOLFOX6 as an adjuvant treatment in colorectal cancer: an economic analysis. Curr Med Res Opin.

[40] Wen F, Yao K, Du ZD, He XF, Zhang PF, Tang RL (2014). Cost-effectiveness analysis of colon cancer treatments from MOSIAC and No. 16968 trials. World J Gastroenterol.

[41] Hsu TC, Wang CC (2019). Cost minimization comparison of oral UFT/leucovorin versus 5-fluorouracil/leucovorin as adjuvant therapy for colorectal cancer in Taiwan. J Comp Eff Res.

[42] Pandor A, Eggington S, Paisley S, Tappenden P, Sutcliffe P (2006). The clinical and cost-effectiveness of oxaliplatin and capecitabine for the adjuvant treatment of colon cancer: systematic review and economic evaluation. Health Technol Assess.

[43] Aballéa S, Boler A, Craig A, Wasan H (2007). An economic evaluation of oxaliplatin for the adjuvant treatment of colon cancer in the United Kingdom (UK). Eur J Cancer.

[44] Aballéa S, Chancellor JV, Raikou M, Drummond MF, Weinstein MC, Jourdan S (2007). Cost-effectiveness analysis of oxaliplatin compared with 5-fluorouracil/leucovorin in adjuvant treatment of stage III colon cancer in the US. Cancer.

[45] Attard CL, Maroun JA, Alloul K, Grima DT, Bernard LM (2010). Cost-effectiveness of oxaliplatin in the adjuvant treatment of colon cancer in Canada. Curr Oncol.

[46] Shiroiwa T, Takeuchi T, Fukuda T, Shimozuma K, Ohashi Y (2012). Cost-effectiveness of adjuvant FOLFOX therapy for stage III colon cancer in Japan based on the MOSAIC trial. Value Health.

[47] Robles-Zurita J, Boyd KA, Briggs AH, Iveson T, Kerr RS, Saunders MP (2018). SCOT: a comparison of cost-effectiveness from a large randomised phase III trial of two durations of adjuvant Oxaliplatin combination chemotherapy for colorectal cancer. Br J Cancer.

[48] Iveson T, Boyd KA, Kerr RS, Robles-Zurita J, Saunders MP, Briggs AH (2019). 3-month versus 6-month adjuvant chemotherapy for patients with high-risk stage II and III colorectal cancer: 3-year follow-up of the SCOT non-inferiority RCT. Health Technol Assess.

[49] Hornberger J, Lyman GH, Chien R, Meropol NJ (2012). A multigene prognostic assay for selection of adjuvant chemotherapy in patients with T3, stage II colon cancer: impact on quality-adjusted life expectancy and costs. Value Health.

[50] Alberts SR, Yu TM, Behrens RJ, Renfro LA, Srivastava G, Soori GS (2014). Comparative economics of a 12-gene assay for predicting risk of recurrence in stage II colon cancer. Pharmacoeconomics.

[51] Jongeneel G, Greuter MJE, van Erning FN, Koopman M, Vink GR, Punt CJA (2021). Model-based effectiveness and cost-effectiveness of risk-based selection strategies for adjuvant chemotherapy in Dutch stage II colon cancer patients. Therap Adv Gastroenterol.

[52] Alarid-Escudero F, Schrag D, Kuntz KM. CDX2 biomarker testing and adjuvant therapy for stage II colon cancer: an exploratory cost-effectiveness analysis. Value Health. 2021. 10.1016/j.jval.2021.07.019 PMC8894795 35227453

[53] Ramsey SD, Andersen MR, Etzioni R, Moinpour C, Peacock S, Potosky A (2000). Quality of life in survivors of colorectal carcinoma. Cancer.

[54] Ness RM, Holmes AM, Klein R, Dittus R (1999). Utility valuations for outcome states of colorectal cancer. Am J Gastroenterol.

[55] Iveson TJ, Kerr RS, Saunders MP, Cassidy J, Hollander NH, Tabernero J (2018). 3 versus 6 months of adjuvant oxaliplatin-fluoropyrimidine combination therapy for colorectal cancer (SCOT): an international, randomised, phase 3, non-inferiority trial. Lancet Oncol.

[56] Aspinall SL, Good CB, Zhao X, Cunningham FE, Heron BB, Geraci M (2015). Adjuvant chemotherapy for stage III colon cancer: relative dose intensity and survival among veterans. BMC Cancer.

[57] Twelves C, Scheithauer W, McKendrick J, Seitz JF, Van Hazel G, Wong A (2012). Capecitabine versus 5-fluorouracil/folinic acid as adjuvant therapy for stage III colon cancer: final results from the X-ACT trial with analysis by age and preliminary evidence of a pharmacodynamic marker of efficacy. Ann Oncol.

[58] Eichler H-G, Abadie E, Breckenridge A, Flamion B, Gustafsson LL, Leufkens H (2011). Bridging the efficacy–effectiveness gap: a regulator's perspective on addressing variability of drug response. Nat Rev Drug Discov.

[59] Ankarfeldt MZ, Adalsteinsson E, Groenwold RH, Ali MS, Klungel OH (2017). A systematic literature review on the efficacy-effectiveness gap: comparison of randomized controlled trials and observational studies of glucose-lowering drugs. Clin Epidemiol.

[60] Figueredo A, Charette ML, Maroun J, Brouwers MC, Zuraw L (2004). Adjuvant therapy for stage II colon cancer: a systematic review from the cancer care Ontario program in evidence-based care’s gastrointestinal cancer disease site group. J Clin Oncol.

[61] Efficacy of adjuvant fluorouracil and folinic acid in B2 colon cancer. International Multicentre Pooled Analysis of B2 Colon Cancer Trials (IMPACT B2) Investigators. J Clin Oncol. 1999;17(5):1356–63. 10334519

[62] Lemmens V, van Steenbergen L, Janssen-Heijnen M, Martijn H, Rutten H, Coebergh JW (2010). Trends in colorectal cancer in the south of the Netherlands 1975–2007: rectal cancer survival levels with colon cancer survival. Acta Oncol.

[63] Renouf D, Kennecke H, Gill S (2008). Trends in chemotherapy utilization for colorectal cancer. Clin Colorectal Cancer.

[64] Tie J, Cohen JD, Lahouel K, Lo SN, Wang Y, Kosmider S (2022). Circulating tumor DNA analysis guiding adjuvant therapy in stage II colon cancer. N Engl J Med.

