# Supplementary material for: Health economic evidence for adjuvant chemotherapy in stage II and III colon cancer: a systematic review
Source: Cost Eff Resour Alloc. 2023 Jan 31;21:11. doi: 10.1186/s12962-023-00422-2 (PMC9887815; doi:10.1186/s12962-023-00422-2)
Supplement: Supplementary file 1 — Additional file 1. Supplementary Material 1. [file 12962_2023_422_MOESM1_ESM.docx]

**Health economic evidence of adjuvant therapy in stage II and III colon cancer: a systematic review**

**Additional file 1**

**Author:** Yat Hang To^1,2^, Peter Gibbs^1,3,4^, Jeanne Tie^1,2,3,4^, Maarten IJzerman^5,6,7^, Koen Degeling^5,6^

**Institutes**

1. Personalised Oncology Division, Walter and Eliza Hall Institute of Medical Research, Melbourne, Australia.
2. Department of Medical Oncology, Peter MacCallum Cancer Centre, Melbourne, Australia.
3. Department of Medical Oncology, Western Health, Melbourne, Australia
4. Faculty of Medicine & Health Sciences, University of Melbourne, Melbourne, Australia
5. Cancer Health Services Research, Centre for Cancer Research, Faculty of Medicine, Dentistry and Health Sciences, University of Melbourne, Melbourne, Australia
6. Cancer Health Services Research, Centre for Health Policy, Melbourne School of Population and Global Health, Faculty of Medicine, Dentistry and Health Sciences, University of Melbourne, Melbourne, Australia
7. Department of Cancer Research, Peter MacCallum Cancer Centre, Melbourne, Australia

**Corresponding author:**

Yat Hang To

Personalised Oncology Division

Walter & Eliza Hall Institute

1G Royal Parade, Parkville VIC 3052, Australia

Email: [To.YatHang@mh.org.au](mailto:To.YatHang@mh.org.au)

ORCID ID: 0000-0002-1497-1700

1. **Search terms**

The search terms used and number of returned results for each individual platform. The initial search was performed on 8^th^ July 2021 with an updated search performed on 10^th^ December 2021.

*Medline (initial search: 8^th^ July 2021)*

| Item | Search | Results |
| --- | --- | --- |
| 1 | exp Colorectal Neoplasms | 211937 |
| 2 | ((colorectal or bowel or colon* or rectum or rectal or sigmoid or CRC) adj3 (cancer* or neoplasm* or tumo?r or malignan* or carcinoma* or adenocarcinoma* or oncolog*)).mp | 284101 |
| 3 | 1 or 2 | 292571 |
| 4 | exp Chemotherapy, adjuvant/ | 42824 |
| 5 | ((adjuvant or surg* or operat* or post* or cur* or early) adj5 (antineoplastic, chemo* or pharmacotherapy* or drug treatment* or drug therap*)).mp | 68588 |
| 6 | 4 or 5 | 105481 |
| 7 | 3 and 6 | 7705 |
| 8 | (cost* adj3(effective* or efficac* or benef* or util* or minim* or conseq* or analys*)).mp | 265783 |
| 9 | health economic.mp | 5226 |
| 10 | (budget impact or budget-impact).mp | 1628 |
| 11 | (economic adj3 (model* or analy* or assess* or evalut* or impact*)).mp | 40353 |
| 12 | 8 or 9 or 10 or 11 | 291899 |
| 13 | 7 and 12 | 88 |
| 14 | limit 13 to English language | 81 |

*Medline (updated search: 10^th^ December 2021)*

| Item | Search | Result |
| --- | --- | --- |
| 1 | exp Colorectal Neoplasms | 217806 |
| 2 | ((colorectal or bowel or colon* or rectum or rectal or sigmoid or CRC) adj3 (cancer* or neoplasm* or tumor or malignan* or carcinoma* or adenocarcinoma* or oncolog*)).mp | 291317 |
| 3 | 1 or 2 | 299946 |
| 4 | exp Chemotherapy, adjuvant/ | 43869 |
| 5 | ((adjuvant or surg* or operat* or post* or cur* or early) adj5 (antineoplastic, chemo* or pharmacotherapy* or drug treatment* or drug therap*)).mp | 70032 |
| 6 | 4 or 5 | 107851 |
| 7 | 3 and 6 | 7916 |
| 8 | (cost* adj3(effective* or efficac* or benef* or util* or minim* or conseq* or analys*)).mp | 289218 |
| 9 | health economic.mp | 5488 |
| 10 | (budget impact or budget-impact).mp | 1716 |
| 11 | (economic adj3 (model* or analy* or assess* or evalut* or impact*)).mp | 41977 |
| 12 | 8 or 9 or 10 or 11 | 315800 |
| 13 | 7 and 12 | 95 |
| 14 | limit 13 to english language | 87 |
| 15 | Limit 14 to dt = 20210808-20211210 | 0 |

*EMBASE (initial search: 8^th^ July 2021)*

| Item | Search | Results |
| --- | --- | --- |
| 1 | exp Colorectal Neoplasms | 31231 |
| 2 | ((colorectal or bowel or colon* or rectum or rectal or sigmoid or CRC) adj3 (cancer* or neoplasm* or tumo?r or malignan* or carcinoma* or adenocarcinoma* or oncolog*)).mp | 448098 |
| 3 | 1 or 2 | 449258 |
| 4 | exp Chemotherapy, adjuvant/ | 60630 |
| 5 | ((adjuvant or surg* or operat* or post* or cur* or early) adj5 (antineoplastic, chemo* or pharmacotherapy* or drug treatment* or drug therap*)).mp | 423684 |
| 6 | 4 or 5 | 471228 |
| 7 | 3 and 6 | 28957 |
| 8 | (cost* adj3(effective* or efficac* or benef* or util* or minim* or conseq* or analys*)).mp | 380387 |
| 9 | health economic.mp | 8251 |
| 10 | (budget impact or budget-impact).mp | 4644 |
| 11 | (economic adj3 (model* or analy* or assess* or evalut* or impact*)).mp | 46786 |
| 12 | 8 or 9 or 10 or 11 | 414209 |
| 13 | 7 and 12 | 459 |
| 14 | limit 13 to English language | 447 |

*EMBASE (updated search: 10th December 2021)*

| Item | Search | Result |
| --- | --- | --- |
| 1 | exp Colorectal Neoplasms | 32037 |
| 2 | ((colorectal or bowel or colon* or rectum or rectal or sigmoid or CRC) adj3 (cancer* or neoplasm* or tumo?r or malignan* or carcinoma* or adenocarcinoma* or oncolog*)).mp | 459637 |
| 3 | 1 or 2 | 460830 |
| 4 | exp Chemotherapy, adjuvant/ | 63010 |
| 5 | ((adjuvant or surg* or operat* or post* or cur* or early) adj5 (antineoplastic, chemo* or pharmacotherapy* or drug treatment* or drug therap*)).mp | 433615 |
| 6 | 4 or 5 | 483084 |
| 7 | 3 and 6 | 29751 |
| 8 | (cost* adj3(effective* or efficac* or benef* or util* or minim* or conseq* or analys*)).mp | 412751 |
| 9 | health economic.mp | 8536 |
| 10 | (budget impact or budget-impact).mp | 4769 |
| 11 | (economic adj3 (model* or analy* or assess* or evalut* or impact*)).mp | 48369 |
| 12 | 8 or 9 or 10 or 11 | 446352 |
| 13 | 7 and 12 | 484 |
| 14 | limit 13 to english language | 471 |
| 15 | limit 14 to em=202127-202149 | 16 |

*Health Technology Assessment Database (initial search: 8^th^ July 2021)*

| Item | Search | Results |
| --- | --- | --- |
| 1 | exp Colorectal Neoplasms | 242 |
| 2 | ((colorectal or bowel or colon* or rectum or rectal or sigmoid or CRC) adj3 (cancer* or neoplasm* or tumo?r or malignan* or carcinoma* or adenocarcinoma* or oncolog*)).mp | 346 |
| 3 | 1 or 2 | 353 |
| 4 | exp Chemotherapy, adjuvant/ | 57 |
| 5 | ((adjuvant or surg* or operat* or post* or cur* or early) adj5 (antineoplastic, chemo* or pharmacotherapy* or drug treatment* or drug therap*)).mp | 16 |
| 6 | 4 or 5 | 72 |
| 7 | 3 and 6 | 11 |
| 8 | (cost* adj3(effective* or efficac* or benef* or util* or minim* or conseq* or analys*)).mp | 2508 |
| 9 | health economic.mp | 59 |
| 10 | (budget impact or budget-impact).mp | 59 |
| 11 | (economic adj3 (model* or analy* or assess* or evalut* or impact*)).mp | 358 |
| 12 | 8 or 9 or 10 or 11 | 2629 |
| 13 | 7 and 12 | 4 |
| 14 | limit 13 to English language | 4 |

*Health Technology Assessment Database (updated search: 10^th^ December 2021)*

| Item | Search | Result |
| --- | --- | --- |
| 1 | exp Colorectal Neoplasms | 242 |
| 2 | ((colorectal or bowel or colon* or rectum or rectal or sigmoid or CRC) adj3 (cancer* or neoplasm* or tumo?r or malignan* or carcinoma* or adenocarcinoma* or oncolog*)).mp | 346 |
| 3 | 1 or 2 | 353 |
| 4 | exp Chemotherapy, adjuvant/ | 57 |
| 5 | ((adjuvant or surg* or operat* or post* or cur* or early) adj5 (antineoplastic, chemo* or pharmacotherapy* or drug treatment* or drug therap*)).mp | 16 |
| 6 | 4 or 5 | 72 |
| 7 | 3 and 6 | 11 |
| 8 | (cost* adj3(effective* or efficac* or benef* or util* or minim* or conseq* or analys*)).mp | 2538 |
| 9 | health economic.mp | 59 |
| 10 | (budget impact or budget-impact).mp | 59 |
| 11 | (economic adj3 (model* or analy* or assess* or evalut* or impact*)).mp | 358 |
| 12 | 8 or 9 or 10 or 11 | 2653 |
| 13 | 7 and 12 | 4 |
| 14 | limit 13 to english language | 4 |
| 15 | limit 14 to yr=Current | 0 |

*National Health Service Health Economic Evaluation Database (initial search: 8^th^ July 2021)*

| Item | Search | Results |
| --- | --- | --- |
| 1 | exp Colorectal Neoplasms | 415 |
| 2 | ((colorectal or bowel or colon* or rectum or rectal or sigmoid or CRC) adj3 (cancer* or neoplasm* or tumo?r or malignan* or carcinoma* or adenocarcinoma* or oncolog*)).mp | 459 |
| 3 | 1 or 2 | 470 |
| 4 | exp Chemotherapy, adjuvant/ | 140 |
| 5 | ((adjuvant or surg* or operat* or post* or cur* or early) adj5 (antineoplastic, chemo* or pharmacotherapy* or drug treatment* or drug therap*)).mp | 40 |
| 6 | 4 or 5 | 180 |
| 7 | 3 and 6 | 21 |
| 8 | (cost* adj3(effective* or efficac* or benef* or util* or minim* or conseq* or analys*)).mp | 15506 |
| 9 | health economic.mp | 162 |
| 10 | (budget impact or budget-impact).mp | 109 |
| 11 | (economic adj3 (model* or analy* or assess* or evalut* or impact*)).mp | 9188 |
| 12 | 8 or 9 or 10 or 11 | 15615 |
| 13 | 7 and 12 | 21 |
| 14 | limit 13 to english language | 20 |

*National Health Service Health Economic Evaluation Database (updated search: 12^th^December 2021)*

| Item | Search | Result |
| --- | --- | --- |
| 1 | exp Colorectal Neoplasms | 415 |
| 2 | ((colorectal or bowel or colon* or rectum or rectal or sigmoid or CRC) adj3 (cancer* or neoplasm* or tumor or malignan* or carcinoma* or adenocarcinoma* or oncolog*)).mp | 459 |
| 3 | 1 or 2 | 470 |
| 4 | exp Chemotherapy, adjuvant/ | 140 |
| 5 | ((adjuvant or surg* or operat* or post* or cur* or early) adj5 (antineoplastic, chemo* or pharmacotherapy* or drug treatment* or drug therap*)).mp | 40 |
| 6 | 4 or 5 | 180 |
| 7 | 3 and 6 | 21 |
| 8 | (cost* adj3(effective* or efficac* or benef* or util* or minim* or conseq* or analys*)).mp | 15536 |
| 9 | health economic.mp | 162 |
| 10 | (budget impact or budget-impact).mp | 109 |
| 11 | (economic adj3 (model* or analy* or assess* or evalut* or impact*)).mp | 9188 |
| 12 | 8 or 9 or 10 or 11 | 15644 |
| 13 | 7 and 12 | 21 |
| 14 | limit 13 to english langugage | 20 |
| 15 | limit 14 to yr=Current | 0 |

1. **CHEERS checklist scoring**

The Consolidated Health Economic Evaluation Reporting Standards (CHEERS) checklist is a published set of recommendations guiding economic evaluation reporting. We adapted the checklist into a report-quality scoring tool, scoring each study according to the proportion of applicable CHEERS items they reported. The detailed results of the scoring is demonstrated below.

|  | Title | Abstract | Background and Objectives | Target populations and subgroups | Setting and Location | Study Perspective | Comparators | Time Horizon | Discount rate | Choice of Health Outcomes | Measurement of Effectiveness | Preference-based outcomes | Resources and Costs | Currency, Price Date, and Conversion | Choice of Model | Assumptions | Analytic Methods | Study Parameters | Incremental Costs and Outcomes | Characterizing Uncertainty | Characterizing Heterogeneity | Findings | Source of Funding | Conflicts of Interest |
| --- | --- | --- | --- | --- | --- | --- | --- | --- | --- | --- | --- | --- | --- | --- | --- | --- | --- | --- | --- | --- | --- | --- | --- | --- |
| Aballea, 2007 | Y | N | Y | Y | Y | Y | Y | Y | Y | Y | Y | Y | Y | Y | Y | Y | Y | N | Y | Y | N/A | Y | Y | Y |
| Aballea, 2007 | Y | N | Y | Y | Y | Y | Y | Y | Y | Y | Y | y | Y | Y | Y | Y | Y | N | Y | Y | N/A | Y | Y | Y |
| Alarid-Escudero, 2021 | Y | Y | Y | Y | Y | Y | Y | Y | Y | Y | Y | Y | Y | Y | Y | Y | Y | Y | Y | Y | Y | Y | Y | Y |
| Alberts, 2014 | Y | N | Y | Y | Y | Y | Y | Y | Y | Y | Y | y | Y | Y | Y | Y | Y | Y | Y | Y | N/A | Y | Y | Y |
| Attard, 2010 | Y | Y | Y | Y | Y | Y | Y | Y | Y | Y | Y | y | Y | Y | N | Y | Y | Y | Y | Y | Y | Y | Y | N |
| Ayvaci, 2013 | Y | Y | Y | Y | Y | Y | Y | Y | Y | Y | Y | y | Y | Y | Y | Y | Y | Y | Y | Y | Y | Y | Y | N |
| Brown, 1994 | Y | Y | Y | Y | N | N | Y | Y | Y | Y | Y | Y | Y | N | Y | Y | Y | N | Y | Y | N/A | Y | N | N |
| Cassidy, 2006 | Y | Y | Y | Y | Y | Y | Y | Y | Y | Y | Y | Y | Y | Y | Y | Y | Y | N | Y | Y | N/A | Y | Y | N |
| Chen,2015 | Y | N | Y | Y | Y | Y | Y | N | N | Y | Y | Y | Y | Y | Y | Y | Y | Y | Y | Y | Y | Y | Y | Y |
| Di Constanzo, 2008 | Y | N | Y | Y | Y | Y | Y | Y | Y | Y | Y | y | Y | Y | N | Y | Y | N | Y | N | N/A | Y | Y | Y |
| Douillard, 2007 | Y | Y | Y | Y | Y | Y | Y | Y | Y | Y | Y | N/A | Y | Y | N | Y | Y | N | Y | N | N/A | Y | Y | N |
| Eggington, 2006 | Y | N | Y | Y | Y | Y | Y | Y | Y | Y | Y | Y | Y | Y | Y | Y | Y | N | Y | Y | N/A | Y | Y | N |
| Goerner, 2009 | Y | N | Y | Y | Y | Y | Y | Y | N | N/A | N/A | N/A | Y | Y | N | Y | Y | Y | Y | Y | Y | Y | N | Y |
| Hanna, 2021 | Y | N | Y | Y | Y | Y | Y | Y | Y | Y | Y | Y | Y | Y | Y | Y | Y | Y | Y | Y | Y | Y | Y | Y |
| Ho, 2016 | Y | N | Y | Y | Y | Y | Y | Y | N | Y | Y | N/A | Y | Y | N | Y | N/A | Y | Y | N | N/A | Y | Y | N |
| Hornberger, 2012 | Y | Y | Y | Y | Y | Y | Y | Y | Y | Y | Y | y | Y | Y | Y | Y | Y | Y | Y | Y | Y | Y | Y | Y |
| Hsu, 2011 | Y | Y | Y | Y | Y | Y | Y | Y | Y | Y | Y | Y | Y | Y | Y | Y | Y | N | Y | Y | N/A | Y | Y | N |
| Hsu, 2019 | Y | Y | Y | Y | Y | Y | Y | N | N | Y | Y | N/A | Y | Y | N | Y | N | Y | Y | N | N/A | Y | Y | Y |
| Iveson, 2019 | N/A | Y | Y | Y | Y | Y | Y | Y | Y | Y | Y | Y | Y | Y | Y | Y | Y | Y | Y | Y | Y | Y | Y | Y |
| Jongeneel, 2020 | Y | Y | Y | Y | Y | Y | Y | Y | Y | Y | Y | Y | Y | Y | Y | Y | Y | Y | Y | Y | Y | Y | Y | Y |
| Jongennel, 2021 | Y | Y | Y | Y | Y | Y | Y | Y | Y | Y | Y | Y | Y | Y | Y | Y | Y | Y | Y | Y | Y | Y | Y | Y |
| Liarosn, 2014 | Y | Y | Y | Y | Y | Y | Y | Y | Y | Y | Y | y | Y | Y | N | Y | Y | N | Y | Y | Y | Y | Y | Y |
| Lerdkiattikorn, 2015 | Y | N | Y | Y | Y | Y | Y | Y | Y | Y | Y | Y | Y | Y | Y | Y | Y | Y | Y | Y | N/A | Y | Y | Y |
| Lin, 2015 | Y | N | Y | Y | Y | Y | Y | Y | Y | Y | Y | Y | Y | Y | N | Y | Y | Y | Y | Y | Y | Y | Y | Y |
| Maniadakis, 2009 | Y | N | Y | Y | Y | Y | Y | Y | N | Y | Y | N/A | Y | Y | N | Y | Y | Y | Y | Y | N/A | Y | Y | Y |
| Michel, 1999 | N | Y | N | Y | Y | Y | Y | Y | Y | Y | Y | N/A | Y | N | Y | Y | Y | Y | Y | Y | N/A | Y | N | N |
| Murad, 1997 | Y | N | Y | Y | Y | Y | Y | Y | N | Y | Y | N/A | Y | Y | N | Y | Y | N | Y | Y | N/A | Y | Y |  |
| Norum, 1997 | Y | Y | Y | Y | Y | Y | Y | Y | Y | Y | Y | Y | Y | Y | N | Y | Y | N | Y | Y | N/A | Y | Y |  |
| Pandor, 2006 | Y | Y | Y | Y | Y | Y | Y | Y | Y | Y | Y | Y | Y | Y | Y | Y | Y | Y | Y | Y | N/A | Y | Y | Y |
| Robles-Zurita, 2018 | Y | Y | Y | Y | Y | Y | Y | Y | Y | Y | Y | Y | Y | Y | Y | Y | Y | Y | Y | Y | Y | Y | Y | Y |
| Shiroiwa, 2009 | Y | Y | Y | Y | Y | Y | Y | Y | Y | Y | Y | Y | Y | Y | Y | Y | Y | Y | Y | Y | N/A | Y | Y | Y |
| Shiroiwa, 2012 | Y | Y | Y | Y | Y | Y | Y | Y | Y | Y | Y | y | Y | Y | Y | Y | Y | N | Y | Y | N/A | Y | Y | Y |
| Smith, 1993 | Y | N | Y | Y | Y | Y | Y | Y | Y | Y | Y | Y | Y | N | N | Y | N | N | Y | Y | N/A | Y | N | N |
| Soni, 2014 | Y | Y | Y | Y | Y | Y | Y | Y | Y | Y | Y | y | Y | Y | Y | Y | Y | Y | Y | Y | Y | Y | Y | Y |
| To, 2021 | Y | Y | Y | Y | Y | Y | Y | Y | Y | Y | Y | y | Y | Y | Y | Y | Y | Y | Y | Y | N/A | Y | Y | Y |
| VanGils, 2015 | Y | N | Y | Y | Y | Y | Y | Y | N | N/A | N/A | N/A | Y | Y | Y | Y | Y | Y | Y | Y | Y | Y | Y | Y |
| Wen, 2014 | Y | Y | Y | Y | Y | Y | Y | Y | N | Y | Y | Y | Y | N | Y | Y | Y | Y | Y | Y | N/A | Y | N | N |
| Xie, 2013 | Y | N | Y | Y | Y | Y | Y | Y | N | N/A | N/A | N/A | Y | Y | Y | Y | N | Y | Y | N | N/A | Y | N | N |
